# Comparative Transcriptome Analysis of Softening and Ripening-Related Genes in Kiwifruit Cultivars Treated with Ethylene

**DOI:** 10.3390/cimb44060177

**Published:** 2022-06-02

**Authors:** Han Ryul Choi, Min Woo Baek, Cheon Soon Jeong, Shimeles Tilahun

**Affiliations:** 1 Department of Horticulture, Kangwon National University, Chuncheon 24341, Korea; hanryul192@kangwon.ac.kr (H.R.C.); minwoo100@kangwon.ac.kr (M.W.B.); 2 Interdisciplinary Program in Smart Agriculture, Kangwon National Uinversity, Chuncheon 24341, Korea; 3 Agriculture and Life Science Research Institute, Kangwon National University, Chuncheon 24341, Korea; 4 Department of Horticulture and Plant Sciences, Jimma University, Jimma 378, Ethiopia

**Keywords:** kiwifruit, transcriptome analysis, gene expression, ethylene treatment, softening

## Abstract

This work presents the transcriptome analysis of green ‘Hayward’ (*Actinidia deliciosa*) and gold ‘Haegeum’ (*Actinidia chinensis*) kiwifruit cultivars after treatment with ethylene for three days at 25 °C. Illumina high-throughput sequencing platform was used to sequence total mRNAs and the transcriptome gene set was constructed by de novo assembly. A total of 1287 and 1724 unigenes were differentially expressed during the comparison of ethylene treatment with control in green ‘Hayward’ and gold ‘Haegeum’, respectively. From the differentially expressed unigenes, 594 and 906 were upregulated, and 693 and 818 were downregulated in the green and gold kiwifruit cultivars, respectively, when treated with ethylene. We also identified a list of genes that were expressed commonly and exclusively in the green and gold kiwifruit cultivars treated with ethylene. Several genes were expressed differentially during the ripening of kiwifruits, and their cumulative effect brought about the softening- and ripening-related changes. This work also identified and categorized genes related to softening and other changes during ripening. Furthermore, the transcript levels of 12 selected representative genes from the differentially expressed genes (DEGs) identified in the transcriptome analysis were confirmed via quantitative real-time PCR (qRT-PCR) to validate the reliability of the expression profiles obtained from RNA-Seq. The data obtained from the present study will add to the information available on the molecular mechanisms of the effects of ethylene during the ripening of kiwifruits. This study will also provide resources for further studies of the genes related to ripening, helping kiwifruit breeders and postharvest technologists to improve ripening quality.

## 1. Introduction

Kiwifruit (*Actinidia* spp.) is a perennial deciduous fruit belonging to the family Actinidiaceae, widely cultivated in 41 countries around the world [1]. In 2019, the global production of kiwifruits was 4.35 million tons, of which the Republic of Korea accounted for 5622 [2]. There are more than 70 different kiwifruit species in the world, and they are classified according to the color of both skin and flesh of the fruit [3]. However, only a few representative cultivars, including green-fleshed *Actinidia deliciosa* and yellow-fleshed *Actinidia chinensis*, have dominated the international commercial market [2]. 

Kiwifruit has been called ‘the king of fruits’ owing to its excellent flavor and outstanding functional substances including minerals, vitamins, and antioxidants [4,5]. Several studies have recommended kiwifruit for diet, anti-cancer effects, relieving stress, and strengthening immunity [6]. Levels of primary and secondary metabolites and the genes encoding their expressions vary depending on genotype, maturity stage, and storage period [2].

Kiwifruit is a climacteric fruit that generates autocatalytic ethylene through the respiration process during ripening [7]; it can be harvested at physiological maturity at an unripe stage [8]. To fulfill consumers’ preference for “ready to eat” kiwifruit, it is imperative to use postharvest techniques such as exogenous ethylene treatment [3]. Exogenous ethylene treatment in kiwifruit has the advantage of rapidly and uniformly ripening the fruit up to the optimal edible stage [9]. The kiwifruit ripening process of involves changes in many gene expressions, biochemical and physiological processes [1].

In recent years, various studies related to ripening and storage of kiwifruit have been reported. Tilahun et al. [3] treated kiwifruit cultivars with exogenous ethylene to determine ripening quality based on sensory evaluation and physicochemical criteria, reporting that gold ‘Haegeum’ and red ‘Hongyang’ kiwifruits attained eating quality on the 2nd day of ripening, whereas green ‘Hayward’ attained eating quality on the 4th day, irrespective of harvest time. The effect of cold storage was also investigated on ripening quality, primary and secondary metabolites, antioxidant activities, and ripening and stress-related genes, to determine biological markers for indication of storability and ripening quality in kiwifruits [2].

Transcriptome profiling analyses related to ripening have been reported for other fruits, including persimmon [10], banana [11], orange [12], watermelon [13] and strawberry [14]. Similarly, the transcript profile of kiwifruit during ripening has been studied. Hydrogen sulfide (H_2_S) treatment can delay the ripening of kiwifruit by regulating cell-wall- and ethylene-related genes [4]. Tilahun et al. [15] reported transcriptome analysis of gold ‘Haeguem’ kiwifruit treated with ethylene to improve postharvest ripening quality. However, comparative transcriptome analysis information has been lacking for the two cultivars in relation to kiwifruit softening and ripening-related changes following ethylene treatment.

In this study, we conducted comparative transcriptome analysis and classified by cultivar the candidate genes related to softening- and ripening-related changes. This enabled our assessment of the differences in postharvest fruit quality, sensory acceptance, and transcriptome profile between naturally ripe and exogenous ethylene-induced ripe kiwifruit during ripening. By analyzing the DEG of kiwifruit cultivars following exogenous ethylene treatment, candidate genes that could be engaged in softening- and ripening-related changes were identified. The expression profiles of 12 selected representative differentially expressed genes were confirmed by quantitative real-time PCR to validate the RNA sequencing results. The transcriptome profile provided by our study will provide useful information on the effects of ethylene treatment on softening- and ripening-related changes in kiwifruit at the genomic level. It could be helpful for further research into genes related to ripening for use in kiwifruit breeding and postharvest technology.

## 2. Materials and Methods

### 2.1. Plant Material and Ethylene Treatment

Two kiwifruit (*Actinidia* spp.) cultivars, green ‘Hayward’ and gold ‘Haegeum’, were used in this study. Fruits were harvested at commercial maturity (170 days after full bloom) [3] on 23 October 2020 in Jangheung, South Korea. Fruits were immediately transferred to the postharvest laboratory at Kangwon University. After careful selection, uniform-sized fruits free from physical defects were treated with exogenous ethylene at 100 μL kg^−1^ [15], in a sealed 62 L container for 3 days at 25 °C. Air in the sealed container was ventilated and distributed by a fan (Coolertec CT8025L12RA-3P, Zhengzhou, China). Four containers were used and 60 fruits were placed in each container. Fruits were regularly inspected and data were collected at 0 d and on the third day; from ten biological replicates for firmness, soluble solids content (SSC), titratable acidity (TA), brix-acid ratio (BAR), and overall acceptability of the fresh fruit; and from five replicates for respiration rate and ethylene production rate. Samples of fruit flesh were also taken for pectin content, polygalacturonase (PG) activity, and transcriptome analysis. All samples were frozen in liquid nitrogen and stored in a deep freezer (−80 °C). Then, samples for analysis of secondary metabolites were freeze-dried with a vacuum freeze dryer (FDT-8650, Operon, Korea) and the dried samples were ground to powder. 

### 2.2. Transcriptome Sequencing

Samples of each cultivar (green, gold) and each treatment (control, ethylene) were sent for sequencing on the third day. Three replicates were used for transcriptome sequencing. RNA sequencing was performed at DNACARE (Seoul, Korea) using HISAT v2.1.0. Total RNA was extracted and pooledin equal volumes from each sample of the control and ethylene-treated groups [16]. Total RNA was isolated from frozen pulp samples with the Robospin Plant TM Kit (GeneAll, Korea) following the manufacturer’s protocol, and genomic DNA was removed with RNA-free DNase I (Sigma, St. Louis, MO, USA). The quality and content of the extracted RNA were measured using a Nano-drop and cDNA was synthesized with oligo d (T)18 primer and SuperScript^®^ III Reverse Transcriptase (Life Technologies, Carlsbad, CA, USA) from 5 μg of total RNA [10]. RNA sequencing was performed using the Illumina high-throughput sequencing platform according to the manufacturer’s protocol (Illumina Inc., San Diego, CA, USA) in the National Instrumentation Center for Environmental Management (NICEM), Seoul National University in Korea [10]. Raw reads were processed using Trimmomatic(v0.38). Then, reads for each sample were mapped to the reference genome (Hongyang v3.0) by HISAT2(v2.0.13) (http://ccb.jhu.edu/software/hisat2/index.shtml accessed on 10 April 2021). The read count of the transcript expression level was calculated using StringTie(v1.3.4d). Differentially expressed genes (DEGs) were putatively identified using DESeq by comparing the transcript levels in control and ethylene treated kiwifruit using two criteria; false discovery rate (FDR) at *p* < 0.05 and |log_2_ fold change| ≥ 1. The flow chart of mRNA processing is indicated in Figure S1.

### 2.3. Identification of DEGs and Functional Enrichment Analysis

To classify all genes into annotated functional subcategories, blast (e-value 1 × 10^−4^) analysis was performed using the Refseq plant protein sequence of UniProt, TAIR, and NCBI as a database. Gene ontology and pathway analysis were performed using BLAST2GO (version 5.2.4) and the InterProScan program targeting Pfam and KEGG databases.

### 2.4. Verification of DEGs by qRT-PCR

Transcript accumulation of *EXPA8*, *EXPA11*, *QRT1*, *ACO1*, *ACO3*, *ACS3*, *ERF061*, *ERF062*, *TLP1*, *LOX1.5*, *LOX6*, *CYP75B1* was evaluated via quantitative real-time RCR (qRT-PCR), as described by [16], using gene-specific primers (Table S1).

### 2.5. Measurement of Firmness, Pectin Content, EIS and Polygalacturonase(PG) Activity

The flesh firmness of kiwifruit was measured by a Rheometer (Sun Scientific Co., Ltd., Tokyo, Japan) from 10 fruits (mean of two measurements per fruit) by a puncture at the equator with a 3 mm diameter round stainless-steel probe with a flat end and a maximum force of 10 kg [17]. The pectin content, EIS, and polygalacturonase (PG) activity of kiwifruit were measured and expressed as described by [3].

### 2.6. Measurement of Soluble Solids Content (SSC), Titratable Acidity (TA), Brix-Acid Ratio (BAR) and Overall Acceptability

Soluble solids content (SSC) from the juice of each fruit was measured using a digital refractometer (Atago Co., Ltd., Tokyo, Japan) at 20 °C. The unit of measurement was expressed in percent [1]. Titratable acidity (TA) was measured using a DL22 food and beverage analyzer (Mettler Toledo Ltd., Zurich, Switzerland). Diluted kiwifruit juice (1 mL juice to 19 mL distilled water) was used for titration by 0.1 N NaOH up to pH 8.1 to obtain TA, expressed as mg of citric acid per kg of fresh kiwifruit weight. BAR was found by dividing the SSC by the titratable acidity [17]. Overall acceptability was identified as the mean value of the subjective scale for flavor, sweetness, chewiness, and appearance during the ripening period [17]. It was evaluated by 10 trained panels of graduate students, and successive digits were assigned to each rating from 1 = bad to 5 = excellent [17].

### 2.7. Weight Loss, Ethylene Production and Respiration Rates

Fresh weight loss was measured as described by [3]. Kiwifruits were weighed before treatment and weighed again after three days to calculate the percentage (%) weight loss during ripening. The ethylene production rate and respiration rate of kiwifruit were measured and expressed as described by [3].

### 2.8. Total Phenolics, Total Flavonoids, and Vitamin C

Total phenolics and total flavonoids contents were measured from freeze-dried kiwifruit samples, according to the methodology implemented previously in our laboratory and described by [18]. Extraction of ascorbic acid was performed according to [19] with some modification. Kiwifruit powder sample (1 g) was extracted by 10 mL 3% (*w*/*v*) meta-phosphoric acid. The sonicated sample was centrifuged (12,578× *g* for 10 min), the liquid layer of extracts was membrane-filtered (0.22 μm) (Advantec, Tokyo, Japan), and analyzed as described by [20]. Meta-phosphoric acid (0.1%) was used as the mobile phase.

### 2.9. Statistical Analysis

The results of the collected quality parameters were analyzed using SPSS 20.0 and are expressed as mean ± SE. The data were subjected to analysis of variance (ANOVA) to determine the significance of differences between cultivars and treatments (*p* < 0.05). Tests for significance between cultivars and treatments were done using a *t*-test.

## 3. Results and Discussion

### 3.1. Assembly and Annotation

The sequencing and mapping results are summarized in parts A and B in Table 1. A total of 72.14 and 64.09 million reads were generated from the green ‘Hayward’ kiwifruit in the control and ethylene libraries, respectively. A total of 51.44 and 52.72 million reads were generated from gold ‘Haegeum’ kiwifruit in the control and ethylene libraries, respectively. From the total reads, 65.60, 59.06, 48.45, and 49.89 million were mapped with a high mapping rate (>84%) to the reference genome (*A. Chinensis* ‘Hong yang’ v3 Genome) from the green control, green ethylene, gold control, and gold ethylene, respectively.

**Figure 1:**
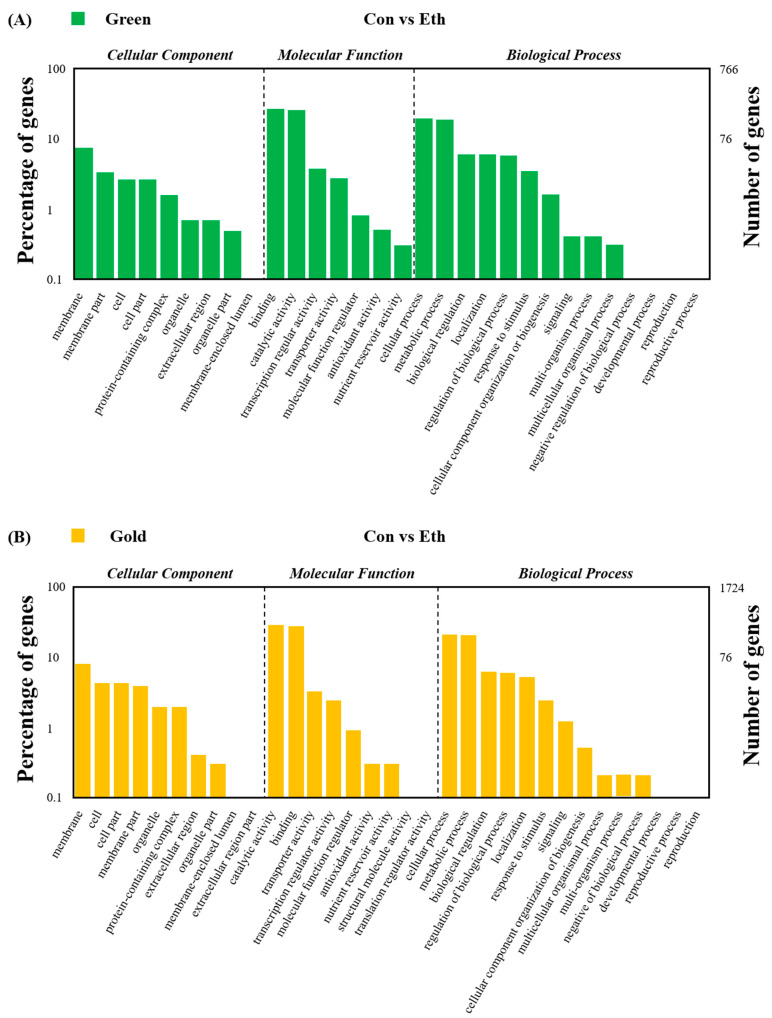
GO classification of the DEGs of control vs. ethylene in (**A**) green ‘Hayward’ and (**B**) gold ‘Haegeum’ kiwifruit.

Figure 1 shows the percentage and number of genes in different gene ontology (GO) classifications of the DEGs of control vs. ethylene in green ‘Hayward’ and gold ‘Haegeum’ kiwifruit. The identified unigenes were classified into three functional categories. The genes in the cellular component were mainly categorized as “membrane” in both cultivars treated with ethylene. Moreover, a higher number of genes in cellular components were mainly involved in “binding” and “catalytic activity” in both cultivars treated with ethylene. The genes in the biological process were enriched “cellular process” and “metabolic process” types in both cultivars treated with ethylene.

We identified a total of 26,130 and 28,605 transcripts during the comparison of control vs. ethylene-treated green ‘Hayward’ and gold ‘Haegeum’ kiwifruit, respectively (part C in Table 1). Differentially expressed genes (DEGs) were compared based on log_2_ fold change and *p* < 0.05 during comparison of control to ethylene. A total of 1287 and 1724 unigenes were differentially expressed during the comparison of control vs. ethylene in green ‘Hayward’ and gold ‘Haegeum’ kiwifruit, respectively.

**Figure 2:**
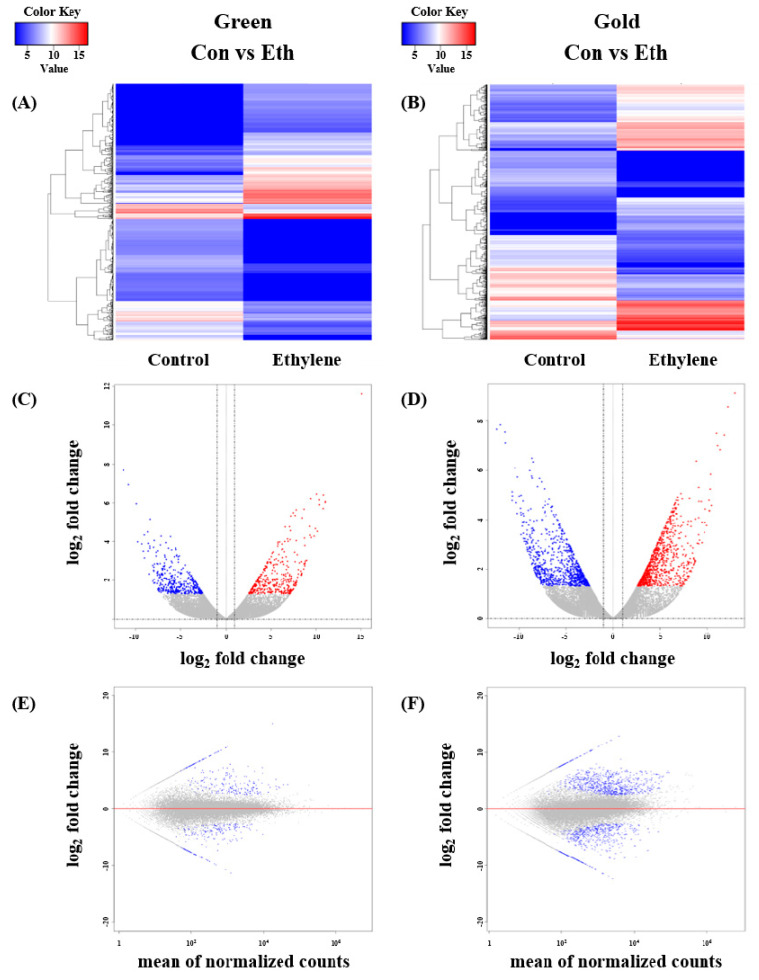
The transcriptional profiles of control vs. ethylene in green ‘Hayward’ and gold ‘Haegeum’ kiwifruit. (**A**,**B**) Heat map visualization. Each gene is represented by a single row. Red indicates relatively high levels, genes with relatively low levels are shown in blue. (**C**,**D**) Volcano plot of the identified genes. The DEGs are shown in red and blue, black indicates genes that were not differentially expressed. (**E**,**F**) MA plot of the identified genes. The DEGs are shown in gray (*p* > 0.05) and blue (*p* < 0.05).

The number of DEGs in the control vs. ethylene treatment groups was compared (Figure 2). Higher DEG numbers were shown in the gold ‘Haegeum’ kiwifruit than in green ‘Hayward’ kiwifruit, as indicated by the heat map, MA plot, and volcano plot.

**Figure 3:**
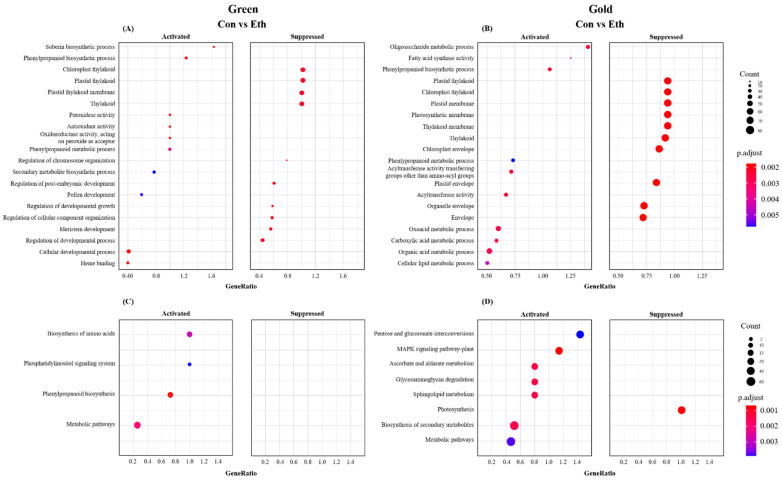
(**A**,**C**) Go enrichment analysis of the differentially expressed genes in green ‘Hayward’ and gold ‘Haegeum’ kiwifruit cultivars. The horizontal axis represents the gene ratio corresponding to the pathway, and the vertical axis represents the pathway name. *p*-adjust values are represented by the color of the points. The gene count in each pathway is indicated by size of point and (**B**,**D**) KEGG pathway enrichment scatter analysis of the differentially expressed genes. The horizontal axis represents the gene ratio corresponding to the pathway, and the vertical axis represents the pathway name. *p*-adjust values are represented by the color of the points. The gene count in each pathway is indicated by size of point.

Go enrichment analysis was applied to investigate the major biological processes affected by ethylene treatment (Figure 3A,B). We identified 20 Go terms that were represented (*p*-adj < 0.01) in response to ethylene treatment. The lowest *p* values for activated biological processes were for “organic acid metabolic process”, “carboxylic acid metabolic process”, “carboxylic acid metabolic process”, “acyltransferase activity”, “acyltransferase activity transferring groups other than amino-acyl groups”, “phenylpropaniod biosynthetic process”, “fatty acid synthase activity”, and “oligosaccharide metabolic process” in green ‘Hayward’ kiwifruit; and “heme binding”, “cellular developmental process”, “oxidoreductase activity, acting on peroxide as acceptor”, “antioxidant activity”, and “peroxidase activity” in gold ‘Haegeum’ kiwifruit. On the other hand, “envelope”, “organelle envelope”, “plastid envelope”, “chloroplast envelope”, “thylakoid”, “thylakoid membrane”, “photosynthetic membrane”, “plastid membrane”, “chloroplast thylakoid”, and “plastid thylakoid” were suppressed in green ‘Hayward’ kiwifruit; “regulation of developmental process”, “meristem development”, “regulation of cellular component organization”, “regulation of developmental growth”, “regulation of post-embryonic development”, “regulation of chromosome organization”, “thylakoid”, “plastid thylakoid”, and “chloroplast thylakoid” were suppressed in gold ‘Haegeum’ kiwifruit. KEGG pathway enrichment analysis of DEGs identified significantly enriched “metabolic pathways” and “biosynthesis of secondary metabolites” in green ‘Hayward’, and it identified “metabolic pathways” and “biosynthesis of amino acids” in gold ‘Haegeum’ kiwifruit (Figure 3C,D).

**Figure 4:**
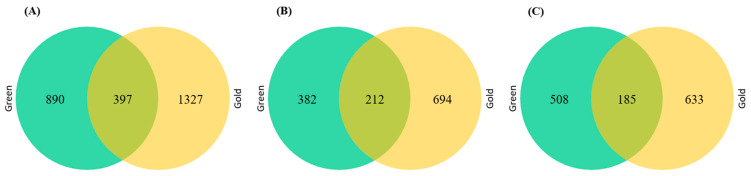
Venn diagram showing the number of differentially expressed genes (log_2_ fold change equal or greater than 1 and *p* < 0.05) total (**A**), upregulated (**B**), and downregulated (**C**), between green ‘Hayward’ and gold ‘Haegeum’ kiwifruit cultivars after 3 days of ripening with ethylene treatment or without ethylene treatment (control) at 25 °C.

The Venn diagram shows 397 commonly expressed genes and 890 and 1327 exclusively expressed genes during the comparison of control vs. ethylene in green ‘Hayward’ and gold ‘Haegeum’ kiwifruit (Figure 4). From the differentially expressed unigenes, 594 (46%) and 906 (53%) were upregulated, and 693 (54%) and 818 (47%) were downregulated in green ‘Hayward’ and gold ‘Haegeum’ kiwifruit, respectively. 

**Figure 5:**
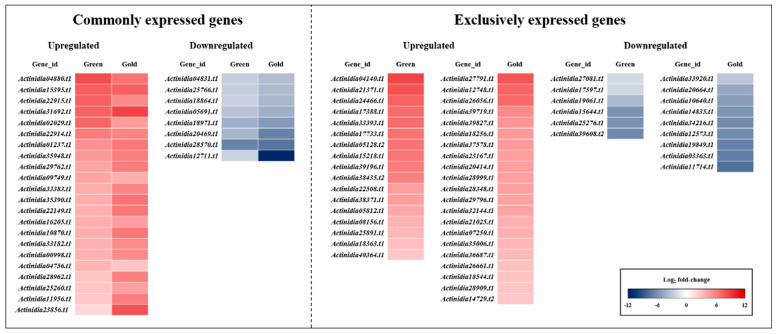
Heat map of commonly and exclusively expressed and identified DEGs related to softening- and ripening-related changes in the comparison of control vs. ethylene-treated green ‘Hayward’ and gold ‘Haegeum’ kiwifruit on the third day of ripening.

Figure 5 shows commonly and exclusively expressed genes of identified DEGs related to softening- and ripening-related changes in the comparison of control vs. ethylene green ‘Hayward’ and gold ‘Haegeum’ kiwifruit by heat map. To identify genes related to softening and ripening, a list of commonly and exclusively expressed genes was aligned (Table 2, Table 3 and Table 4). In addition, some of the identified genes were briefly discussed in comparison with ripening-related parameters. 

### 3.2. Firmness, Total Pectin, EIS, PG Activity, and Related Genes

**Figure 6:**
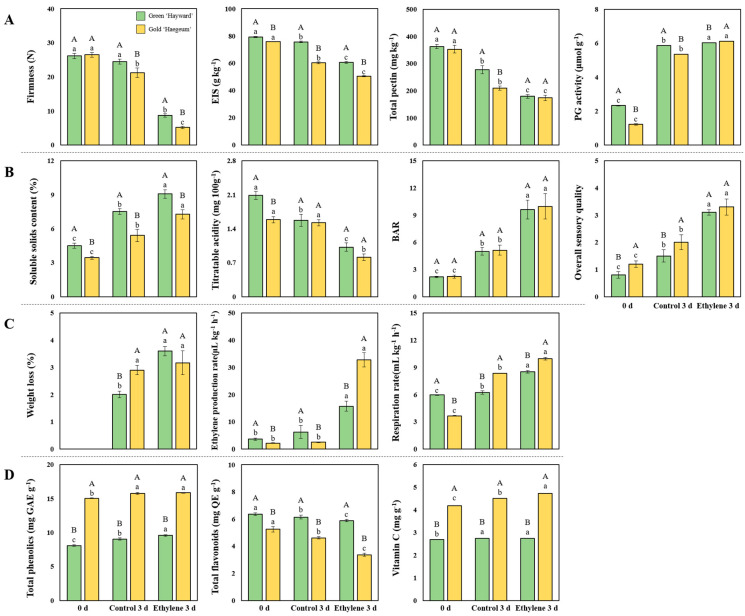
Firmness, EIS, total pectin and PG activity (**A**); soluble solids content (SSC), titratable acidity (TA), Brix/acid ratio (BAR) and overall sensory quality (**B**); weight loss, ethylene production and respiration rates (**C**); total phenolics, total flavonoids and vitamin C (**D**) of green ‘Hayward’ and gold ‘Haegeum’ kiwifruit on day 0 and third day of ripening at 25 °C with ethylene treatment or without treatment (control). Data are presented as a mean ± standard errors in 10 replicates for firmness, SSC, TA, BAR and overall sensory quality; and in 5 replicates for the other parameters. The bars with different upper-case letters indicate a significant difference (*p* < 0.05) between cultivars, the bars with different lower-case letters indicate a significant difference (*p* < 0.05) between treatments.

Firmness and firmness-related parameters are the most important indicators that can determine the quality of fruit [3]. Firmness, total pectin, and EIS were shown at 0 d and on the third day of ripening at 25 °C with or without ethylene treatment (Figure 6A). Firmness, total pectin, and EIS tended to decrease as the ripening period proceeded, and in particular, significant differences were observed in the ethylene treatment group on the third day. Tilahun et al. [3] reported that 5–10 N firmness values for kiwifruit cultivars can fulfill customers’ preferences. In this study, eating quality (5–10 N) after ethylene treatment was attained in green ‘Hayward’ and gold ‘Haegeum’ kiwifruit on the third day, irrespective of cultivars. Concurrently, a significant reduction in EIS and total pectin and a significant increase in PG activity were confirmed in ethylene-treated fruit of both cultivars on the third day. The solubilization of pectin can explain the decrease in total pectin and EIS due to the increase in PG activity as the ripening proceeded in kiwifruit cultivars [17].

The solubilization of pectin polysaccharides in fruit is achieved by the interactive activities of several enzymes such as polygalacturonase (PG), pectate lyase (PL), pectinesterase (PE), pectin acetylesterase (PAE) and beta-galactosidase (b-gal) [21,22]. In this study, the genes that encode *probable pectate lyase 8*, *probable pectate lyase 18*, and *polygalacturonase-like* were commonly upregulated in both cultivars (Table 2). In addition, the genes that encode *probable pectinesterase 53*, *pectin acetylesterase 12*, *beta-galactosidase 13-like*, *beta-galactosidase BG1-like precursor*, and *polygalacturonase At1g48100-like isoform X1* were exclusively upregulated in green ‘Hayward’ kiwifruit (Table 3). In contrast, the genes that encode *pectin acetylesterase 8*, *beta-galactosidase-like*, *beta-galactosidase 17-like*, *beta-galactosidase-like isoform X1*, *probable pectate lyase 8 isoform X1*, *probable pectate lyase 18 isoform X1*, and *polygalacturonase At1g48100* were exclusively upregulated in gold ‘Haegeum’ kiwifruit (Table 3). Consistent with this study, Tilahun et al. [15] reported that the genes that encode *polygalacturonase*, *pectate lyase*, *pectin acetlyesterase*, and *beta-galacturonase* were upregulated due to ethylene treatment in gold ‘Haegeum’ kiwifruit (*Actinidia chinensis*). In this study, the gene encoding *pectinesterase QRT1-like*, involved in the progress of pectin degradation, was commonly upregulated in both cultivars. Consistent with this study, Guo et al. [23] reported that the gene expression of pectinesterase QRT was cultivar-dependent and much higher in ‘Fengzao’ than ‘Kyoho’ grape cultivars. In addition, Zhu et al. [13] reported that protein inhibitors can synchronize the action of pectinesterase, and the gene encoding *pectinesterase inhibitor* was downregulated in two watermelon cultivars during ripening. In this study, the genes that encode *probable pectinesterase/pectinesterase inhibitors 12* and *probable pectinesterase/pectinesterase inhibitor 34* were exclusively downregulated in green ‘Hayward’ and gold ‘Haegeum’ kiwifruit, respectively. *Alpha-xylosidase 1-like*, which plays an essential role in cell wall modification [24], was exclusively upregulated in green ‘Hayward’ kiwifruit. Endoglucanase is an enzyme that loosens the network of xyloglucan-cellulose and hydrolyzes the internal sites of the cellulose chain within the cell wall [25]. In this study, the gene encoding *endoglucanase 24* was commonly downregulated in both cultivars. Furthermore, the gene encoding *endoglucanse 25-like* was exclusively downregulated in gold ‘Haegeum’ kiwifruit. Xyloglucan endotransglucosylase/hydrolase protein participates in cell wall construction by cleaving and linking the xyloglucan polymer that stabilizes the cellulose-hemicellulose framework [15]. In this study, the genes encoding *xyloglucan endotransglucosylase/hydrolase protein 22-like* and *probable xyloglucan endotransglucosylase/hydrolase protein 30* were exclusively upregulated in green ‘Hayward’ kiwifruit. Then, the upregulation of *xyloglucan endotransglucosylase/hydrolase protein 22-like* and *probable xyloglucan endotransglucosylase/hydrolase protein 30* genes could lead to the softening and ripening of kiwifruit. Expansins may play essential roles in cell wall disassembly and precede the action of various cell wall hydrolases [26]. In this study, the genes that encode *expansin-A1*, *expansin-A8-like*, and *expansin-A11* were commonly upregulated in both cultivars. The gene encoding *expansin-A4-like* was exclusively upregulated in gold ‘Haegeum’ kiwifruit. Consistent with this study, Palapol et al. [27] reported that the expression of expansin genes in durian fruit pulp was hastened by ethylene, and ethylene promoted the expression of genes such as DzEXP1 and DzEXP2. The cellulose synthase A catalytic subunit is involved in secondary cell wall formation [28]. In this study, the genes that encode *cellulose synthase A catalytic subunit 2* and *cellulose synthase A catalytic subunit 3* were exclusively downregulated in gold ‘Haegeum’ kiwifruit. Tilahun et al. [15] reported that the gene encoding *cellulose synthase A catalytic subunit 8-like* was downregulated in gold kiwifruit. Malladi et al. [29] and Cao et al. [30] reported that a gene encoding *COBRA* plays an essential role in the regulation of cell wall architecture in fruit. The gene encoding *protein COBRA-like* was exclusively downregulated in green ‘Hayward’ kiwifruit, consistent with Cao et al. [30], who reported the expression of the COBRA gene declined rapidly during ripening in tomatoes. The gene that encodes *shikimate hyroxycinnamoyl transferase* is involved in forming lignin, a significant component of stone cells in pear fruit [31]. In this study, the gene encoding *shikimate O-hydroxycinnamoyltransferase-like* was exclusively upregulated in gold ‘Haegeum’ kiwifruit. The results could be explained by the response of the enzyme to faster ripening after ethylene treatment.

### 3.3. SSC, TA, BAR and Sensory Evaluation and the Related Genes

In this study, both cultivars showed a tendency to increase SSC, BAR, and sensory evaluation scores during the ripening process compared to immediately after harvest. Significant differences were observed, especially on the third day after exogenous ethylene treatment (Figure 6B and Figure S2). Conversely, in both cultivars, titratable acidity during the ripening process tended to decrease compared to immediately after harvest, and a significant difference was observed, especially on the third day after exogenous ethylene treatment.

*Beta-amylase* is a very important enzyme in the starch degradation process because it produces maltose by cleaving the starch chain. As a result, it affects the sweetness of ripe fruit [15,32]. In this study, the genes encoding *beta-amylase 3* and *chloroplastic* were commonly upregulated in both cultivars. Chen et al. [33] reported that *beta-amylase 3* was upregulated in ethylene-treated fruit in African Pride atemoya, suggesting that ethylene treatment might accelerate starch degradation. Chen et al. [34] suggested that sucrose synthase is the key enzyme catalyzing the process of sucrose degradation of kiwifruit. In this study, the gene encoding *sucrose synthase-like* was exclusively downregulated in green ‘Hayward’ kiwifruit. *Phosphoenolpyruvate carboxylase* is the critical enzyme in organic acid biosynthesis in plants, and might be linked to the conversion of organic acids into sugars [15,35]. In this study, the genes encoding *phosphoenolpyruvate carboxylase 4 isoform X2* and *phosphoenolpyruvate carboxylase, housekeeping isozyme* were exclusively downregulated after ethylene treatment due to the reduction of acidity in gold ‘Haegeum’ kiwifruit. In general, *lipoxygenase* is known to be related to fruit ripening quality characteristics such as aroma development in kiwifruit [36]. In this study, the genes encoding *lipoxygenase 6, chloroplastic* and *probable linoleate 9S-lipoxygenase 5 isoform X2* were commonly downregulated in both cultivars. Consistent with this study, Zhang et al. [36] reported the gene expression levels of *LOX 2*, *LOX 3*, *LOX 4*, and *LOX 6* showed a tendency to decrease during kiwifruit ripening and may contribute to producing aroma after ethylene treatment. *Methanol O-anthraniloyltransferase* is an enzyme solely responsible for the production of O-methyl anthranilate, a compound with aroma and flavor in the grapefruit [37]. In this study, the gene encoding *methanol O-anthraniloyltransferase-like* was commonly upregulated in both cultivars. This implies that O-methyl anthranilate can directly influence the aroma and flavor of kiwifruit after ethylene treatment. Zhang et al. [38] and Tilahun et al. [15] reported that ribose-5-phosphate isomerase is essential in the glycolysis and TCA cycle of plants. In this study, the gene encoding *probable ribose-5-phosphate isomerase 2* was exclusively upregulated in gold ‘Haeguem’ kiwifruit. The results imply that the enzyme reaction could lead to faster ripening after ethylene treatment compared to the control.

### 3.4. Ethylene Production and Respiration Rates and Related Genes

Figure 6C shows that the weight loss, ethylene production, and respiration rates were the highest in ethylene-treated green ‘Hayward’ and gold ‘Haegeum’ kiwifruit compared to the values at 0 d and on the third day of the control. Consistent with our results, Tilahun et al. [3] reported a tendency to increase SSC and respiration rate, but a tendency to decrease titratable acidity was observed after exogenous ethylene treatment of kiwifruit. The results may imply that the reduction in TA could be due to the change of organic acid into sugars during ripening. 

S-adenosyl methionine (SAM) synthase is an enzyme that converts methionine to SAM in the ethylene pathway [39]. Aminocyclopropane-1-carboxylic acid (ACC) synthase is involved in the conversion of SAM to ACC [40]. ACC oxidase is an enzyme that catalyzes the conversion of ACC to ethylene [41]. In this study, the genes that encode *1-aminocyclopropane-1-carboxylate synthase 3*, *1-aminocyclopropane-1-carboxylate oxidase*, and *1-aminocyclopropane-1-carboxylate oxidase* were commonly upregulated in both cultivars. The genes that encode *1-aminocyclopropane-1-carboxylate oxidase 5*, and *probable S-adenosylmethionine-dependent methyltransferase At5g38100* were exclusively upregulated in green ‘Hayward’ kiwifruit. The genes that encode *1-aminocyclopropane-1-carboxylate synthase-like*, *1-aminocyclopropane-1-carboxylate oxidase 1 isoform X1*, *S-adenosylmethionine synthase 1*, and *S-adenosylmethionine synthase 3* were also exclusively upregulated in gold ‘Haegeum’ kiwifruit. This suggests that exogenous ethylene application induces more ethylene biosynthesis and thus may increase respiration rate, biochemical changes, color changes, and softening (Figure 6C). *Ethylene response factors (ERFs)*, which play a pivotal role in plant responses to biotic or abiotic stresses, are involved in the ethylene signaling and response pathway [42]. In this study, *ethylene-responsive transcription factor ERF 061-like* and *ethylene-responsive transcription factor ERF 062* were commonly upregulated in both cultivars. The gene encoding *ethylene-responsive transcription factor 2-like* was exclusively upregulated in green ‘Hayward’ kiwifruit. The genes that encode *ethylene-responsive transcription factor ERF073*, *ethylene-responsive transcription factor ERF118*, and *ethylene-responsive transcription factor TINY-like* were exclusively upregulated in gold ‘Haegeum’ kiwifruit. Consistent with this study, Tilahun et al. [10] reported that increased levels in expression of ethylene-related gene families such as *ERF3*, *ERF10*, *ERF18*, *ERF 23*, and *ERF 24*, *ERS1 ethylene receptor*, and *ethylene-responsive transcription factor RAP2-11*, correlated with ripening and softening in astringent persimmon. Ethylene action is achieved by regulating ethylene receptors and triggering signal transduction reactions, and ultimately by controlling relevant gene expression in the fruit [16]. It has been shown that upregulation of ethylene-related gene families after exogenous ethylene treatment has led to higher respiration rates and faster ripening. 

### 3.5. Stress-Related Genes Due to Ethylene Treatment

Secondary metabolites are involved in plant defense and responses against biotic and abiotic stresses [43]. These compounds, such as polyphenols and terpenoids, are also responsible for the fruits’ organoleptic, color, and nutritional characteristics [1]. In this study, total phenolics, flavonoids, and vitamin C were significantly different after exogenous ethylene treatment in green ‘Hayward’ and gold ‘Haegeum’, respectively (Figure 6D). Generally, total phenolics and vitamin C contents were higher in gold ‘Haegeum’ than in green ‘Hayward’ kiwifruit. Conversely, total flavonoid content was higher in green ‘Hayward’ than in gold ‘Haegeum’ kiwifruit.

The gene that encodes *Aldehyde oxidase GLOX-like*, which might play a defense mechanism role in attacking pathogens in grapevines, was commonly upregulated in both cultivars [44]. The gene encoding *chitinase 1-like*, the enzyme that degrades fungal cell wall components, resulting in inhibited fungal growth [45], was commonly upregulated in both cultivars. *Endochitinase*, one of the pathogenesis-related proteins, plays a vital role in plant defense mechanisms against fungal pathogens [46]. In this study, the gene encoding *basic endochitinase* was commonly upregulated in both cultivars. Moreover, the genes encoding *endochitinase-like* and *endochitinase EP3* were exclusively upregulated in green ‘Hayward’ kiwifruit. Glucan endo-1,3-beta-glucosidase may play an essential role in degrading fungal cell wall polysaccharides. In this study, the genes encoding *glucan endo-1,3-beta-glucosidase, acidic-like*, and *glucan endo-1,3-beta-glucosidase*, *basic vacuolar isoform-like* were exclusively upregulated in green ‘Hayward’, and *probable glucan endo-1,3-beta-glucosidase A6* and *glucan endo-1,3-beta-glucosidase 14* were exclusively upregulated in gold ‘Haegeum’ kiwifruit. Consistent with this study, Wurms et al. [47] reported that the expression levels of the gene encoding *glucan endo-1,3-beta-glucosidase* increased in postharvest ripe rot disease in gold ‘Hort 16A’ kiwifruit. Pathogenesis-related protein 4 has powerful antifungal activity against plant pathogens such as *Trichoderma harzianum*, *Fusarium culmorum, F. graminearum*, and *B. cinerea* [46]. In this study, the gene encoding *pathogenesis-related protein 4* was commonly upregulated in both cultivars. The gene encoding *pathogenesis-related protein PR-4-like* was exclusively upregulated in gold ‘Haegeum’ kiwifruit. Bai et al. [48] reported that pathogenesis-related protein 4 is involved in defense responses against *B.dothidea* in *Malus domestica* apple. *Thaumatin-like protein (TLP)* is one of the protein families and plays a vital role in plant defense mechanisms against various biotic and abiotic stresses [49]. In this study, the gene encoding *thaumatin-like protein* was commonly upregulated in both cultivars. Choi et al. [42] reported that *thaumatin-like protein 1* protects tissues from pathogen infection in peach. The GDSL esterase/lipase protein, which plays an important role in plant defense and growth, is a multifunctional hydrolase and has many functions in secondary metabolism, abiotic stress, morphogenesis, seed development, and pathogen defense [50,51]. In this study, the gene encoding *GDSL esterase/lipase* was commonly upregulated in both cultivars, implying that the exogenous application of ethylene could stimulate the defense mechanism due to stresses. The gene encoding *cinnamoyl-CoA reductase-like SNL6*, an enzyme that resists pathogenic infection, was also exclusively downregulated in gold ‘Haegeum’ treated with ethylene [52]. Tilahun et al. [15] reported that Cinnamoyl-CoA reductase could stimulate lignin biosynthesis in kiwifruit due to stresses such as exogenous ethylene treatment and low temperature. And Giordano et al. [53] reported that caffeoyl-CoA O-methyltransferase could be responsible for anthocyanin methylation activity under drought stress conditions in grape berries. In this study, the gene encoding *caffeoyl-CoA O-methyltransferase* was commonly downregulated in both cultivars, and the gene encoding *probable caffeoyl-CoA O-methyltransferase At4g26220 isoform X3* was exclusively downregulated in green ‘Hayward’ kiwifruit. The *transcription factor MYB1R1* enhances drought tolerance by regulating water loss in potatoes [54]. In this study, the gene encoding *transcription factor MYB41* was commonly downregulated in both cultivars. This implies that MYB1R1 functions as a transcription factor involved in stress-related genes. In addition, Chen et al. [55] reported that WRKY transcription factors play vital roles in regulating stress responses in plants. In this study, the genes encoding *probable WRKY transcription factor 70* were commonly downregulated in both cultivars. This implies that the downregulation of the genes encoding the above enzymes could be the response of the kiwifruit to stress caused by the exogenous ethylene treatment. We identified the genes encoding the enzymes involved in the flavonoid biosynthesis pathway, such as *flavonol synthase/flavanone 3-hydroxylase-like*, *flavonoid 3′-monooxygenase*, *flavonoid 3-O-glucosyltransferase*, *flavonol sulfotransferase*, *flavonoid 3′,5′-hydroxylase*, *flavanone 3-hydroxylase*, *flavoprotein-ubiquinone oxidoreductase*, *putative UDP-glucose flavonoid 3-O-glucosyltransferase 3*, *leucoanthocyanidin dioxygenase*, and *glutathione-S-transferase*, in the transcriptome data [56]. In this study, the genes encoding *flavonoid 3′-monooxygenase-like isoform X2*, *putative UDP-glucose flavonoid 3-O-glucosyltransferase 3*, and *flavonol sulfotransferase-like* were commonly downregulated in both cultivars. Moreover, the gene encoding *flavonoid 3′5′-hydroxylase 2* was exclusively upregulated, and the genes encoding *electron transfer flavoprotein-ubiquinone oxidoreductase*, *mitochondrial isoform X1* and *flavonol synthase/flavanone 3-hydroxylase-like* were exclusively downregulated in green ‘Hayward’ kiwifruit. In addition, the gene encoding *glutathione S-transferase 3-like* was exclusively upregulated in gold ‘Haegeum’ kiwifruit, and the genes encoding *leucoanthocyanidin dioxygenase-like* and *glutathione S-transferase U17* were exclusively downregulated in gold ‘Haegeum’ kiwifruit. Glutathione-S-transferase is essential in transporting flavonoids synthesized in the cytosol to vacuoles and other locations in plants [56]. Kobayashi et al. [57] reported that UDP-glucose flavonoid 3-O-glucosyltransferase plays an essential role in anthocyanin biosynthesis in the grape berry. The present study shows that the genes related to the flavonoid biosynthesis pathway interacted with the stress resistance mechanism after exogenous ethylene treatment. *Laccase* is a well-known redox enzyme that oxidizes various phenols and aroma substances based on oxidation-reduction reactions [58]. In this study, the gene encoding *laccase-15-like* was commonly upregulated in both cultivars. Yihui et al. [59] reported that laccase-related genes such as *LAC7* and *LAC9* in ‘Red Delicious’ and *LAC7*, *LAC9*, *LAC14, LAC15,* and *LAC16* in ‘Cortland’ were upregulated after 4 and 7 months of storage in apples. Thioredoxin plays an important role in plants‘ tolerance of oxidative stress [60]. The genes encoding *thioredoxin-like protein CXXS1* in green ‘Hayward’, and those for *thioredoxin-like protein CITRX1, chloroplastic* and *thioredoxin-like 1–2, chloroplastic* in gold ‘Haegeum’ kiwifruit were exclusively upregulated. Consistent with this study, [60] reported that expression of *thioredoxin* such as *MaTrx6* and *MaTrx12* was upregulated in banana fruit after ethylene treatment.

### 3.6. Verification of DEGs by qRT-PCR

**Figure 7:**
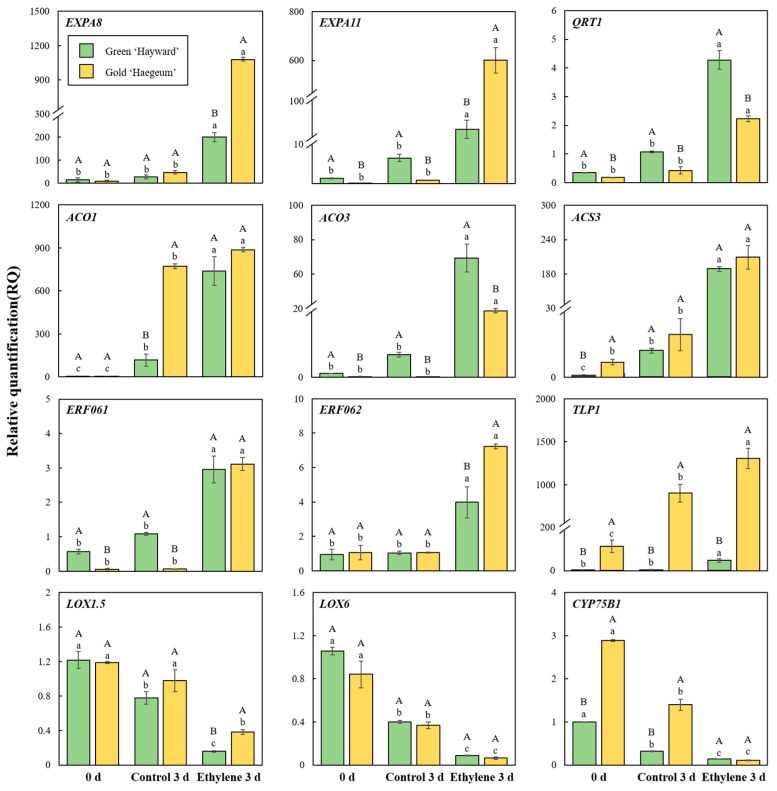
qRT-PCR transcript accumulation of the selected DEGs of green ‘Hayward’ and gold ‘Haegeum’ kiwifruit for the comparison of fruit on day 0 and third day of ripening at 25 °C with ethylene treatment or without treatment (control). Vertical bars represent standard errors of the means (*n* = 3). The bars with different upper-case letters indicate a significant difference (*p* < 0.05) between cultivars, whereas the bars with different lower-case letters indicate a significant difference (*p* < 0.05) between treatments. The names of genes are indicated: *EXPA8 (expansin-A8-like)*, *EXPA11 (expansin-A11)*, *QRT1 (pectinesterase QRT1)*, *ACO1 (1-aminocyclopropane-1-carboxylate oxidase 1)*, *ACO3 (1-aminocyclopropane-1-carboxylate oxidase 3)*, *ACS3 (1-aminocyclopropane-1-carboxylate synthase 3)*, *ERF061 (ethylene-responsive transcription factor ERF061)*, *ERF062 (ethylene-responsive transcription factor ERF062)*, *TLP1 (thaumatin-like protein)*, *LOX1.5 (probable linoleate 9S-lipoxygenase 5 isoform X2)*, *LOX6 (lipoxygenase 6, chloroplastic)*, and *CYP75B1 (flavonoid 3’-monooxygenase-like isoform X2)*.

To validate the RNA-Seq results, we selected 12 representative genes related to softening and ripening-related changes from the differentially expressed genes (Figure 7). Transcript accumulation of the selected representative genes (EXPA8, EXPA11, QRT1, ACO1, ACO3, ACS3, ERF061, ERF062, TLP1, LOX1.5, LOX6, CYP75B1) from the differentially expressed genes was evaluated by quantitative qRT-PCR [42]. The gene expression results by qRT-PCR were consistent with those obtained from RNA-seq for the 12 observed genes (Figure 7, Table 2). Based on these results, the coincided expression patterns of these representative genes in the RNA-seq assay and qRT-PCR suggest the reliability of the RNA-seq data.

## 4. Conclusions

This study reported the comparative transcriptome analysis of kiwifruit cultivars after treatment with ethylene for three days at 25 °C. We compared ethylene-treated fruit with the control, to study the candidate genes related to softening and other changes during ripening. Several genes were expressed commonly and exclusively in the green and gold kiwifruit cultivars treated with ethylene. The findings also showed that various genes were expressed differentially during the ripening of kiwifruits with exogenous ethylene application, and the cumulative effect brought softening- and ripening-related changes. In addition, this work identified and categorized genes related to softening and other changes during ripening. The data obtained from the present study will add to the information available on the molecular mechanisms of the effects of ethylene during kiwifruit ripening. This study will provide resources for further study of the genes related to ripening, for kiwifruit breeding and postharvest technologists to improve ripening quality.

## Figures and Tables

**Table 1:** Summary of raw and trimmed data (A) and mapping rate (B) and differentially expressed genes (DEGs) during the comparison of control vs. ethylene-treated green ‘Hayward’ and gold ‘Haegeum’ kiwifruit cultivars (C).

**(A)**					
**Sample**	**Raw Data**		**Trimmed Data**		
	**Total reads**	**Total read bases (bp)**	**Total reads**	**Total read bases (bp)**	**%**
**Green_Con**	85,610,346	13,520,193,606	85,610,346	12,683,777,637	93.8%
**Green_Eth**	75,655,556	11,974,790,146	75,655,556	11,152,550,066	93.1%
**Gold_Con**	61,430,214	6,204,451,614	58,940,256	5,834,133,639	94.0%
**Gold_Eth**	61,777,836	6,239,561,436	59,494,040	5,873,712,144	94.1%
**(B)**					
**Sample**	**Progressed read**	**Mapped reads**	**Properly paired reads**		**Mapping rate**
**Green_Con**	85,610,346	72,135,718	65,600,622		84.3%
**Green_Eth**	75,665,556	64,094,382	59,061,836		84.7%
**Gold_Con**	58,940,256	51,437,355	48,447,084		87.3%
**Gold_Eth**	59,494,040	52,721,368	49,888,316		88.6%
**(C)**					
	**Total transcripts**	***p* < 0.05, ǀlog_2_ fold changeǀ ≥ 1**			
		**UP**	**DOWN**		**TOTAL**
**Green Con vs. Eth**	26,130	594	693		1287
**Gold Con vs. Eth**	28,605	906	818		1724

**Table 2:** List of DEGs common to green ‘Hayward’ and gold ‘Haegeum’ kiwifruit in the comparison of ethylene treated vs. control.

Gene_id	Gene Descriptions	Log_2_ Fold Change		*p* Value	
		Green	Gold	Green	Gold
**Upregulated**					
Actinidia04880.t1	*expansin-A1*	8.41	6.65	0.000	0.024
Actinidia15395.t1	*aldehyde oxidase GLOX-like*	7.59	7.52	0.003	0.016
Actinidia22915.t1	*chitinase 1-like*	7.50	5.58	0.000	0.000
Actinidia31692.t1	*pathogenesis-related protein 4*	7.44	8.77	0.005	0.000
Actinidia02029.t1	*thaumatin-like protein*	7.24	4.74	0.000	0.002
Actinidia22914.t1	*basic endochitinase*	6.30	5.83	0.000	0.002
Actinidia01237.t1	*methanol O-anthraniloyltransferase-like*	5.23	6.43	0.000	0.002
Actinidia35948.t1	*probable pectate lyase 8*	5.02	6.13	0.000	0.002
Actinidia29762.t1	*expansin-A11*	4.33	6.18	0.000	0.002
Actinidia09749.t1	*1-aminocyclopropane-1-carboxylate oxidase 3*	4.31	3.93	0.000	0.011
Actinidia33383.t1	*laccase-15-like*	3.98	5.91	0.039	0.020
Actinidia35390.t1	*pectinesterase QRT1-like*	3.97	6.68	0.000	0.000
Actinidia22149.t1	*ethylene-responsive transcription factor ERF061-like*	3.95	6.41	0.000	0.000
Actinidia16205.t1	*probable pectate lyase 18*	3.89	4.61	0.008	0.022
Actinidia10870.t1	*beta-amylase 3, chloroplastic*	3.89	6.42	0.000	0.003
Actinidia33182.t1	*ethylene-responsive transcription factor ERF062*	3.83	5.51	0.014	0.005
Actinidia00998.t1	*expansin-A8-like*	3.78	5.50	0.000	0.002
Actinidia04756.t1	*cellulose synthase A catalytic subunit 2*	3.71	2.80	0.000	0.034
Actinidia28962.t1	*polygalacturonase-like*	2.84	6.10	0.027	0.006
Actinidia25260.t1	*1-aminocyclopropane-1-carboxylate oxidase 1*	2.84	4.52	0.029	0.004
Actinidia11956.t1	*1-aminocyclopropane-1-carboxylate synthase 3*	2.65	6.17	0.009	0.000
Actinidia23856.t1	*GDSL esterase/lipase*	1.93	8.06	0.045	0.000
**Downregulated**					
Actinidia04831.t1	*flavonoid 3′-monooxygenase-like isoform X2*	−2.66	−3.54	0.035	0.013
Actinidia25766.t1	*lipoxygenase 6, chloroplastic*	−2.69	−3.63	0.016	0.012
Actinidia18864.t1	*putative UDP-glucose flavonoid 3-O-glucosyltransferase 3*	−2.46	−3.90	0.034	0.004
Actinidia05691.t1	*probable linoleate 9S-lipoxygenase 5 isoform X2*	−3.29	−4.35	0.001	0.002
Actinidia18971.t1	*LOW QUALITY PROTEIN: flavonol sulfotransferase-like*	−4.29	−5.46	0.003	0.000
Actinidia20469.t1	*endoglucanase 24*	−4.05	−6.74	0.022	0.022
Actinidia28570.t1	*probable WRKY transcription factor 70*	−6.74	−7.44	0.023	0.031
Actinidia12711.t1	*transcription factor MYB41*	−2.42	−11.42	0.024	0.000

**Table 3:** List of DEGs exclusive to green ‘Hayward’ kiwifruit in the comparison of ethylene treated vs. control.

Gene_id	Gene Descriptions	Log_2_ Fold Change	*p* Value
**Upregulated**			
Actinidia04140.t1	*xyloglucan endotransglucosylase/hydrolase protein 22-like*	8.68	0.000
Actinidia21371.t1	*probable pectinesterase 53*	8.16	0.001
Actinidia24466.t1	*flavonoid 3′,5* *′-hydroxylase 2*	7.40	0.006
Actinidia17388.t1	*endochitinase-like*	6.97	0.011
Actinidia33393.t1	*endochitinase EP3*	6.96	0.000
Actinidia17733.t1	*pectin acetylesterase 12*	6.77	0.021
Actinidia05128.t2	*thioredoxin-like protein CXXS1*	6.64	0.009
Actinidia15218.t1	*1-aminocyclopropane-1-carboxylate oxidase 5*	6.52	0.000
Actinidia39196.t1	*glucan endo-1,3-beta-glucosidase, acidic-like*	6.22	0.038
Actinidia38435.t2	*probable S-adenosylmethionine-dependent methyltransferase At5g38100*	6.03	0.032
Actinidia22508.t1	*beta-galactosidase BG1-like precursor*	4.69	0.034
Actinidia38371.t1	*alpha-xylosidase 1-like*	4.64	0.009
Actinidia05812.t1	*probable xyloglucan endotransglucosylase/hydrolase protein 30*	4.22	0.000
Actinidia08156.t1	*polygalacturonase At1g48100-like isoform X1*	3.65	0.030
Actinidia25891.t1	*ethylene-responsive transcription factor 2-like*	3.42	0.006
Actinidia18363.t1	*pathogenesis-related protein PR-4-like*	3.18	0.002
Actinidia40364.t1	*glucan endo-1,3-beta-glucosidase, basic vacuolar isoform-like*	2.73	0.028
**Downregulated**			
Actinidia27081.t1	*electron transfer flavoprotein-ubiquinone oxidoreductase, mitochondrial isoform X1*	−2.03	0.036
Actinidia17597.t1	*protein COBRA-like*	−2.20	0.023
Actinidia19061.t1	*flavonol synthase/flavanone 3-hydroxylase-like*	−3.78	0.016
Actinidia15644.t1	*sucrose synthase-like*	−5.89	0.034
Actinidia25276.t1	*probable caffeoyl-CoA O-methyltransferase At4g26220 isoform X3*	−5.95	0.015
Actinidia39608.t2	*probable pectinesterase/pectinesterase inhibitor 12*	−6.39	0.000

**Table 4:** List of DEGs exclusive to gold ‘Haeguem’ kiwifruit in the comparison of ethylene treated vs. control.

Gene_id	Gene Descriptions	Log_2_ Fold Change	*p* Value
**Upregulated**			
Actinidia27791.t1	*polygalacturonase At1g48100*	7.99	0.019
Actinidia12748.t1	*thioredoxin-like protein CITRX1, chloroplastic*	7.29	0.041
Actinidia26056.t1	*beta-galactosidase 17-like*	7.08	0.021
Actinidia39719.t1	*beta-galactosidase-like isoform X1*	5.64	0.000
Actinidia39827.t1	*ethylene-responsive transcription factor ERF118*	5.05	0.010
Actinidia18256.t1	*shikimate O-hydroxycinnamoyltransferase-like*	4.81	0.001
Actinidia37578.t1	*probable pectate lyase 8 isoform X1*	4.77	0.007
Actinidia23167.t1	*expansin-A4-like*	4.68	0.004
Actinidia20414.t1	*ethylene-responsive transcription factor TINY-like*	4.63	0.032
Actinidia28999.t1	*probable glucan endo-1,3-beta-glucosidase A6*	4.56	0.019
Actinidia28348.t1	*1-aminocyclopropane-1-carboxylate synthase-like*	4.55	0.016
Actinidia29796.t1	*1-aminocyclopropane-1-carboxylate oxidase 1 isoform X1*	4.44	0.005
Actinidia32144.t1	*beta-galactosidase-like*	4.39	0.002
Actinidia21025.t1	*ethylene-responsive transcription factor ERF073*	3.79	0.006
Actinidia07250.t1	*glucan endo-1,3-beta-glucosidase 14*	3.78	0.010
Actinidia35006.t1	*thioredoxin-like 1–2, chloroplastic*	3.47	0.010
Actinidia36687.t1	*probable ribose-5-phosphate isomerase 2*	3.27	0.019
Actinidia26661.t1	*S-adenosylmethionine synthase 1*	3.05	0.022
Actinidia18544.t1	*probable pectate lyase 18 isoform X1*	2.91	0.041
Actinidia28909.t1	*S-adenosylmethionine synthase 3*	2.82	0.035
Actinidia14729.t2	*pectin acetylesterase 8-like*	2.73	0.042
**Downregulated**			
Actinidia33920.t1	*leucoanthocyanidin dioxygenase-like*	−3.07	0.044
Actinidia20664.t1	*cellulose synthase A catalytic subunit 3*	−4.71	0.001
Actinidia10640.t1	*cellulose synthase A catalytic subunit 2*	−5.30	0.017
Actinidia14835.t1	*phosphoenolpyruvate carboxylase 4 isoform X2*	−5.75	0.000
Actinidia34216.t1	*probable pectinesterase/pectinesterase inhibitor 34*	−6.32	0.000
Actinidia12573.t1	*phosphoenolpyruvate carboxylase, housekeeping isozyme*	−6.32	0.001
Actinidia19849.t1	*glutathione S-transferase U17*	−6.79	0.028
Actinidia03363.t1	*cinnamoyl-CoA reductase-like SNL6*	−7.03	0.031
Actinidia11714.t1	*endoglucanase 25-like*	−7.75	0.019

## Data Availability

All data sets are available upon reasonable request from the corresponding author.

## Supplementary Materials

The following supporting information can be downloaded at: https://www.mdpi.com/article/10.3390/cimb44060177/s1, Figure S1: Flow chart of mRNA processing; Table S1: Selected genes and primers used for validation of gene expression during qRT-PCR; Figure S2: Green ‘Hayward’ and Gold ‘Haegeum’ kiwifruit cultivars at 0 d and after 3 days of ripening with ethylene treatment or without ethylene treatment (control) at 25 °C.

## References

[1] Choi H.R., Baek M.W., Cheol L.H., Jeong C.S., Tilahun S. (2022). Changes in metabolites and antioxidant activities of green ‘Hayward’and gold ‘Haegeum’ kiwifruits during ripening with ethylene treatment. Food Chem..

[2] Choi H.R., Baek M.W., Tilahun S., Jeong C.S. (2022). Long-term cold storage affects metabolites, antioxidant activities, and ripening and stress-related genes of kiwifruit cultivars. Postharvest Biol. Technol..

[3] Tilahun S., Choi H.R., Lee Y.M., Choi J.H., Baek M.W., Hyok K., Park S.M., Jeong C.S. (2020). Ripening quality of kiwifruit cultivars is affected by harvest time. Sci. Hortic..

[4] Lin X., Yang R., Dou Y., Zhang W., Du H., Zhu L., Chen J. (2020). Transcriptome analysis reveals delaying of the ripening and cell-wall degradation of kiwifruit by hydrogen sulfide. J. Sci. Food Agric..

[5] Hunter D.C., Skinner M.A., Ferguson A.R. (2016). Kiwifruit and health. Fruits, Vegetables, and Herbs.

[6] Meena N.K., Baghel M., Jain S.K., Asrey R. (2018). Postharvest biology and technology of kiwifruit. Postharvest Biology and Technology of Temperate Fruits.

[7] Chiaramonti N., Barboni T. (2010). Relationship between the physicochemical parameters and the ethylene emission during cold storage of kiwifruits. Int. J. Food Sci. Technol..

[8] Schroder R., Atkinson R.G. (2006). Kiwifruit cell walls: Towards an understanding of softening?. N. Z. J. For. Sci..

[9] Lim S., Lee J.G., Lee E.J. (2017). Comparison of fruit quality and GC–MS-based metabolite profiling of kiwifruit ‘Jecy green’: Natural and exogenous ethylene-induced ripening. Food Chem..

[10] Tilahun S., Park K.C., Choi I.Y., Jeong C.S. (2019). Transcriptome analysis of astringent ‘Cheongdo-Bansi’persimmon fruit treated with ethylene for removal of astringency. Postharvest Biol. Technol..

[11] Asif M.H., Lakhwani D., Pathak S., Gupta P., Bag S.K., Nath P., Trivedi P.K. (2014). Transcriptome analysis of ripe and unripe fruit tissue of banana identifies major metabolic networks involved in fruit ripening process. BMC Plant Biol..

[12] Yu K., Xu Q., Da X., Guo F., Ding Y., Deng X. (2012). Transcriptome changes during fruit development and ripening of sweet orange (*Citrus sinensis*). BMC Genom..

[13] Zhu Q., Gao P., Liu S., Zhu Z., Amanullah S., Davis A.R., Luan F. (2017). Comparative transcriptome analysis of two contrasting watermelon genotypes during fruit development and ripening. BMC Genom..

[14] Wang Q.H., Zhao C., Zhang M., Li Y.Z., Shen Y.Y., Guo J.X. (2017). Transcriptome analysis around the onset of strawberry fruit ripening uncovers an important role of oxidative phosphorylation in ripening. Sci. Rep..

[15] Tilahun S., Choi H.R., Kwon H., Park S.M., Park D.S., Jeong C.S. (2020). Transcriptome analysis of ‘Haegeum’ gold kiwifruit following ethylene treatment to improve postharvest ripening quality. Agronomy.

[16] Tilahun S., Heo J.Y., Jeong C.S. (2017). Quality and expression of ethylene response genes of ‘Daebong’ persimmon fruit during ripening at different temperatures. Postharvest Biol. Technol..

[17] Choi H.R., Tilahun S., Lee Y.M., Choi J.H., Baek M.W., Jeong C.S. (2019). Harvest time affects quality and storability of kiwifruit (*Actinidia* spp.): Cultivars during long-term cool storage. Sci. Hortic..

[18] Baek M.W., Choi H.R., Solomon T., Jeong C.S., Lee O.H., Tilahun S. (2021). Preharvest methyl jasmonate treatment increased the antioxidant activity and glucosinolate contents of hydroponically grown pak choi. Antioxidants.

[19] Krupa T., Latocha P., Liwińska A. (2011). Changes of physicochemical quality, phenolics and vitamin C content in hardy kiwifruit (*Actinidia arguta* and its hybrid) during storage. Sci. Hortic..

[20] Tilahun S., Choi H.R., Baek M.W., Cheol L.H., Kwak K.W., Park D.S., Solomon T., Jeong C.S. (2021). Antioxidant Properties, γ-Aminobutyric Acid (GABA) Content, and Physicochemical Characteristics of Tomato Cultivars. Agronomy.

[21] Fabi J.P., Broetto S.G., Silva S.L.G.L.D., Zhong S., Lajolo F.M., do Nascimento J.R.O. (2014). Analysis of papaya cell wall-related genes during fruit ripening indicates a central role of polygalacturonases during pulp softening. PLoS ONE.

[22] Xiao L., Li T., Jiang G., Jiang Y., Duan X. (2019). Cell wall proteome analysis of banana fruit softening using iTRAQ technology. J. Proteom..

[23] Guo D.L., Xi F.F., Yu Y.H., Zhang X.Y., Zhang G.H., Zhong G.Y. (2016). Comparative RNA-Seq profiling of berry development between table grape ‘Kyoho’and its early-ripening mutant ‘Fengzao’. BMC Genom..

[24] Wu T., Cao J. (2008). Differential gene expression of tropical pumpkin (*Cucurbita moschata* Duchesne) bush mutant during internode development. Sci. Hortic..

[25] Jara K., Castro R.I., Ramos P., Parra-Palma C., Valenzuela-Riffo F., Morales-Quintana L. (2019). Molecular insights into FaEG1, a strawberry endoglucanase enzyme expressed during strawberry fruit ripening. Plants.

[26] Sane V.A., Chourasia A., Nath P. (2005). Softening in mango (*Mangifera indica* cv. Dashehari) is correlated with the expression of an early ethylene responsive, ripening related expansin gene, MiExpA1. Postharvest Biol. Technol..

[27] Palapol Y., Kunyamee S., Thongkhum M., Ketsa S., Ferguson I.B., van Doorn W.G. (2015). Expression of expansin genes in the pulp and the dehiscence zone of ripening durian (*Durio zibethinus*) fruit. J. Plant Physiol..

[28] Kotake T., Aohara T., Hirano K., Sato A., Kaneko Y., Tsumuraya Y., Takatsuji H., Kawasaki S. (2011). Rice *Brittle culm* 6 encodes a dominant-negative form of CesA protein that perturbs cellulose synthesis in secondary cell walls. J. Exp. Bot..

[29] Malladi A. (2020). Molecular physiology of fruit growth in apple. Hortic. Rev..

[30] Cao Y., Tang X., Giovannoni J., Xiao F., Liu Y. (2012). Functional characterization of a tomato COBRA-likegene functioning in fruit development and ripening. BMC Plant Biol..

[31] Ma C., Zhang H., Li J., Tao S., Qiao X., Korban S.S., Zhang S., Wu J. (2017). Genome-wide analysis and characterization of molecular evolution of the HCT gene family in pear (*Pyrus bretschneideri*). Plant Syst. Evol..

[32] Setiabudi E., Meitha K., Dwivany F.M. (2021). In silico characterization and comparison of the fruit ripening related beta-amylase (BAM) gene family in banana genome A and B. Indones. J. Biotechnol..

[33] Chen J., Duan Y., Hu Y., Li W., Sun D., Hu H., Xie J. (2019). Transcriptome analysis of atemoya pericarp elucidates the role of polysaccharide metabolism in fruit ripening and cracking after harvest. BMC Plant Biol..

[34] Chen C., Yuan Y., Zhang C., Li H., Ma F., Li M. (2017). Sucrose phloem unloading follows an apoplastic pathway with high sucrose synthase in Actinidia fruit. Plant Sci..

[35] Moing A., Rothan C., Svanella L., Just D., Diakou P., Raymond P., Gaudillère J.P., Monet R. (2000). Role of phosphoenolpyruvate carboxylase in organic acid accumulation during peach fruit development. Physiol. Plant..

[36] Zhang B., Yin X.R., Li X., Yang S.L., Ferguson I.B., Chen K.S. (2009). Lipoxygenase gene expression in ripening kiwifruit in relation to ethylene and aroma production. J. Agric. Food Chem..

[37] Wang J., Luca V.D. (2005). The biosynthesis and regulation of biosynthesis of Concord grape fruit esters, including ‘foxy’ methylanthranilate. Plant J..

[38] Zhang R.G., Andersson C.E., Savchenko A., Skarina T., Evdokimova E., Beasley S., Arrowsmith C.H., Edwards A.M., Joachimiak A., Mowbray S.L. (2003). Structure of *Escherichia coli* ribose-5-phosphate isomerase: A ubiquitous enzyme of the pentose phosphate pathway and the Calvin cycle. Structure.

[39] Pattyn J., Vaughan-Hirsch J., Van de Poel B. (2021). The regulation of ethylene biosynthesis: A complex multilevel control circuitry. New Phytol..

[40] Kende H. (1989). Enzymes of ethylene biosynthesis. Plant Physiol..

[41] John P. (1991). How plant molecular biologists revealed a surprising relationship between two enzymes, which took an enzyme out of a membrane where it was not located, and put it into the soluble phase where it could be studied. Plant Mol. Biol. Report..

[42] Choi H.R., Jeong M.J., Baek M.W., Choi J.H., Lee H.C., Jeong C.S., Tilahun S. (2021). Transcriptome Analysis of Pre-Storage 1-MCP and High CO_2_-Treated ‘Madoka’ Peach Fruit Explains the Reduction in Chilling Injury and Improvement of Storage Period by Delaying Ripening. Int. J. Mol. Sci..

[43] Leitzmann C. (2016). Characteristics and health benefits of phytochemicals. Complementary Med. Res..

[44] Balestrini R., Ghignone S., Quiroga G., Fiorilli V., Romano I., Gambino G. (2020). Long-term impact of chemical and alternative fungicides applied to Grapevine *cv* Nebbiolo on Berry Transcriptome. Int. J. Mol. Sci..

[45] Kitajima S., Sato F. (1999). Plant pathogenesis-related proteins: Molecular mechanisms of gene expression and protein function. J. Biochem..

[46] Selitrennikoff C.P. (2001). Antifungal proteins. Appl. Environ. Microbiol..

[47] Wurms K., Cui W., Ah-Chee A., Rees-George J., Bublin M., Breiteneder H. (2011). Down Regulation of Putative Defence-associated Transcripts Correlates with Ripe Rot Symptoms on Kiwifruit (*Actinidia chinensis*). J. Phytopathol..

[48] Bai S., Dong C., Li B., Dai H. (2013). A PR-4 gene identified from *Malus domestica* is involved in the defense responses against *Botryosphaeria dothidea*. Plant Physiol. Biochem..

[49] Cao J., Lv Y., Hou Z., Li X., Ding L. (2016). Expansion and evolution of thaumatin-like protein (TLP) gene family in six plants. Plant Growth Regul..

[50] Li H., Han X., Qiu W., Xu D., Wang Y., Yu M., Wang Y., Zhuo R. (2019). Identification and expression analysis of the GDSL esterase/lipase family genes, and the characterization of SaGLIP8 in *Sedum alfredii* Hance under cadmium stress. PeerJ.

[51] Ni P.Y., Ji X.R., Guo D.L. (2020). Genome-wide identification, characterization, and expression analysis of GDSL-type esterases/lipases gene family in relation to grape berry ripening. Sci. Hortic..

[52] Singh A., Singh S., Singh R., Kumar S., Singh S.K., Singh I.K. (2021). Dynamics of Zea mays transcriptome in response to a polyphagous herbivore, *Spodoptera litura*. Funct. Integr. Genom..

[53] Giordano D., Provenzano S., Ferrandino A., Vitali M., Pagliarani C., Roman F., Cardinale F., Castellarin S.D., Schubert A. (2016). Characterization of a multifunctional caffeoyl-CoA O-methyltransferase activated in grape berries upon drought stress. Plant Physiol. Biochem..

[54] Shin D., Moon S.J., Han S., Kim B.G., Park S.R., Lee S.K., Yoon H.J., Lee H.E., Kwon H.B., Baek D.W. (2011). Expression of StMYB1R-1, a novel potato single MYB-like domain transcription factor, increases drought tolerance. Plant Physiol..

[55] Chen L., Song Y., Li S., Zhang L., Zou C., Yu D. (2012). The role of WRKY transcription factors in plant abiotic stresses. Biochim. Biophys. Acta (BBA)-Gene Regul. Mech..

[56] Liang W., Ni L., Carballar-Lejarazú R., Zou X., Sun W., Wu L., Yuan X., Mao Y., Huang W., Zou S. (2019). Comparative transcriptome among *Euscaphis konishii* Hayata tissues and analysis of genes involved in flavonoid biosynthesis and accumulation. BMC Genom..

[57] Kobayashi S., Ishimaru M., Ding C.K., Yakushiji H., Goto N. (2001). Comparison of UDP-glucose: Flavonoid 3-O-glucosyltransferase (UFGT) gene sequences between white grapes (*Vitis vinifera*) and their sports with red skin. Plant Sci..

[58] Oh T.G., Jo J.A., Lee S.J. (2021). Evaluation of time–temperature integrator for indicating the ripeness of kiwifruit in plastic container at home. J. Food Sci..

[59] Yihui G., Song J., Du L., Vinqvist M., Palmer L.C., Fillmore S., Pang X., Zhang Z. (2018). Characterization of laccase from apple fruit during postharvest storage and its response to diphenylamine and 1-methylcyclopropene treatments. Food Chem..

[60] Wu F., Li Q., Yan H., Zhang D., Jiang G., Jiang Y., Duan X. (2016). Characteristics of three thioredoxin genes and their role in chilling tolerance of harvested banana fruit. Int. J. Mol. Sci..

