# Supplementary material for: Comparative Transcriptome Analysis of Softening and Ripening-Related Genes in Kiwifruit Cultivars Treated with Ethylene
Source: Curr Issues Mol Biol. 2022 Jun 2;44(6):2593–613. doi: 10.3390/cimb44060177 (PMC9221576; doi:10.3390/cimb44060177)
Supplement: Supplementary file 1 [file cimb-44-00177-s001.zip › cimb-1753166-supplementary.pdf]

current issues in
molecular biology

Comparative Transcriptome Analysis of Softening and Ripening-related Genes in Kiwifruit Cultivars Treated with Ethylene

Han Ryul Choi [1,2], Min Woo Baek [1,2], Cheon Soon Jeong [1,2,*] and Shimeles Tilahun [1,3,4,*]

[1] Department of Horticulture, Kangwon National University, Chuncheon 24341, Korea; hanryul192@kangwon.ac.kr (H.R.C); minwoo100@kangwon.ac.kr (M.W.B)
[2] Interdisciplinary Program in Smart Agriculture, Kangwon National Uinversity, Chuncheon 24341, Korea
[3] Agriculture and Life Science Research Institute, Kangwon National University, Chuncheon 24341, Korea
[4] Department of Horticulture and Plant Sciences, Jimma University, Jimma 378, Ethiopia; e-mail@e-mail.com
* Correspondence: jeongcs@kangwon.ac.kr (C.S.J); shimeles@kangwon.ac.kr (S.T.); Tel.: +82-033-250-6409 (C.S.J.)

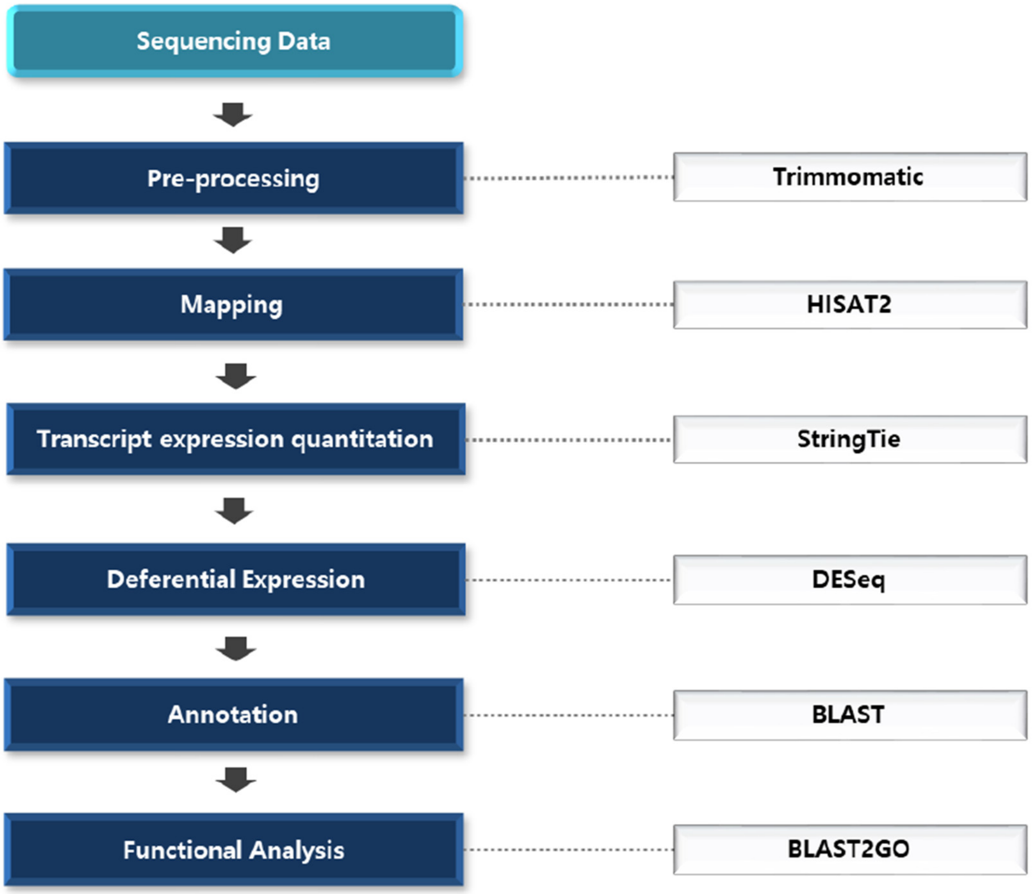

Figure S1. Flow chart of mRNA processing.

Table S1. Selected genes and primers used for validation of gene expression during qRT-PCR.

Gene_id	Gene Descriptions	Primer Sequence 5′-3′- Forward	Reverse
Acti-nidia00998.t1	*expansin-A8-like (EXPA8)*	CAATCAATGGCCACTCC-TACTT	CACCCTGACTTTGATCCCTTG
Acti-nidia29762.t1	*expansin-A11 (EXPA11)*	GTGGGCAGTGCTACAAGATTA	TCATAGTTTGGTGGGCAGAAG
Acti-nidia35390.t1	*pectinesterase QRT1 (QRT1)*	CAGACTCCAA-GCCCTACATTTC	CCGCCGTCCTTTCCTTTATC
Acti-nidia25260.t1	*1-aminocyclopropane-1-carboxylate oxidase 1 (ACO1)*	CCGGCTCCCAAACTCTTA-TATC	ATTCAAATCTGGGACCCTTCTC
Acti-nidia09749.t1	*1-aminocyclopropane-1-carboxylate oxidase 3 (ACO3)*	CGATCTCGACGACGAA-TATAGG	CCAGTCCCAGATTCTCACATAG
Acti-nidia11956.t1	*1-aminocyclopropane-1-carboxylate synthase 3 (ACS3)*	GCTCGCTCTGTTCCAAGATTA	CGGATCGAAGCTCACTTTGT
Acti-nidia22149.t1	*ethylene-responsive transcription factor ERF061 (ERF061)*	CCATCAAACACCGCGATTTC	CCTCTGTCTCACTCCTCTGTAT
Acti-nidia33182.t1	*ethylene-responsive transcription factor ERF062 (ERF062)*	CCACAACCAACATCAC-CAAAG	ACCATCTCAGATCCAGCTTTAC
Acti-nidia02029.t1	*thaumatin-like protein (TLP1)*	CCGGTGGATGCAATAATCCT	GTAAGCATCTGGGCACCTATC
Acti-nidia05691.t1	*probable linoleate 9S-lipoxygenase 5 isoform X2 (LOX1.5)*	GGAGGAATGG-GATCGTGTTTAT	CAGAAGACCCTCCAAGGATTG
Acti-nidia25766.t1	*lipoxygenase 6, chloroplastic (LOX6)*	GAGCCTGAGTACGA-GAAGTTTATT	AGTGTCTTGGATCGCCATTAC

Actinidia04831.t1	*flavonoid 3′-monooxygenase-like isoform X2 (CYP75B1)*	CAGCCCAATTCCTCAAGGT	GCGAACACAAGGTCCTGATA

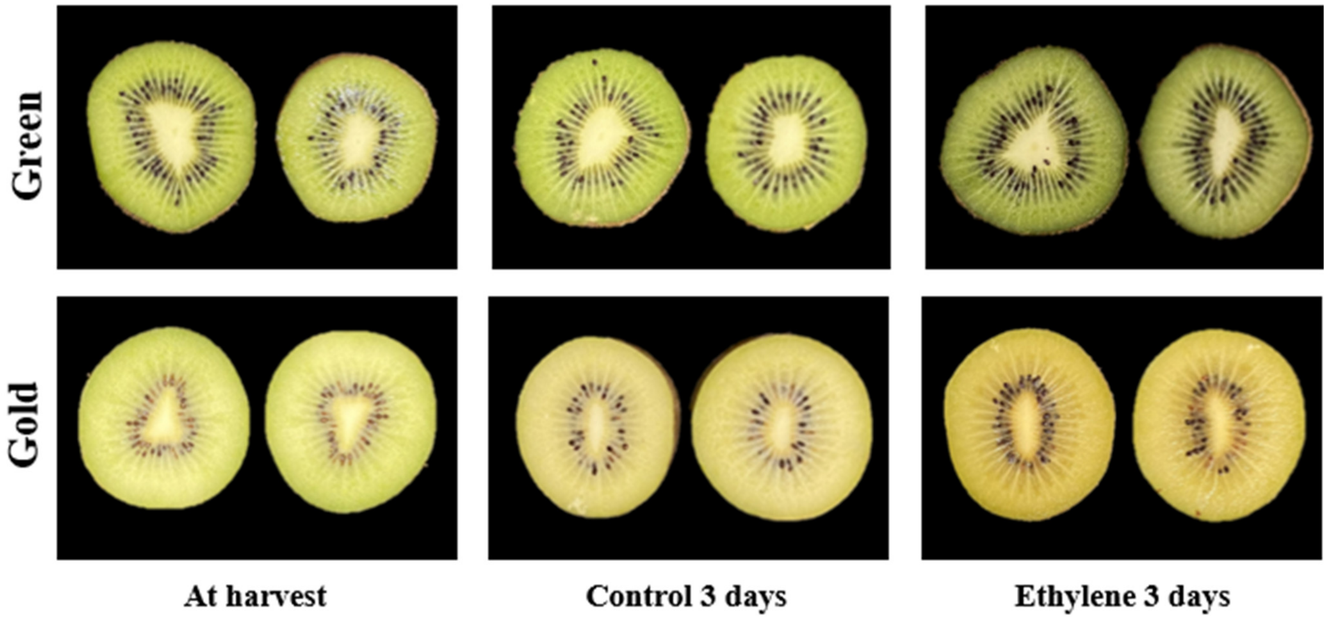

Figure S2. Green 'Hayward' and Gold 'Haegeum' kiwifruit cultivars at harvest and after 3 days of ripening with ethylene treatment or without ethylene treatment (control) at 25 °C.
